# The Diverse Piscidin Repertoire of the European Sea Bass (*Dicentrarchus labrax*): Molecular Characterization and Antimicrobial Activities

**DOI:** 10.3390/ijms21134613

**Published:** 2020-06-29

**Authors:** Carolina Barroso, Pedro Carvalho, Carla Carvalho, Nuno Santarém, José F. M. Gonçalves, Pedro N. S. Rodrigues, João V. Neves

**Affiliations:** 1 i3S—Instituto de Investigação e Inovação em Saúde, Universidade do Porto, 4200-135 Porto, Portugal; carla.carvalho@ibmc.up.pt (C.C.); santarem@ibmc.up.pt (N.S.); prodrigu@ibmc.up.pt (P.N.S.R.); jneves@ibmc.up.pt (J.V.N.); 2 Iron and Innate Immunity, IBMC—Instituto de Biologia Celular e Molecular, Universidade do Porto, 4200-135 Porto, Portugal; 3 Programa Doutoral em Biologia Molecular e Celular (MCbiology), ICBAS—Instituto de Ciências Biomédicas Abel Salazar, Universidade do Porto, 4050-313 Porto, Portugal; 4 ICBAS—Instituto de Ciências Biomédicas Abel Salazar, Universidade do Porto, 4050-313 Porto, Portugal; pecarvalho@icbas.up.pt (P.C.); jfmg@icbas.up.pt (J.F.M.G.); 5 Parasite Disease, IBMC—Instituto de Biologia Celular e Molecular, Universidade do Porto, 4200-135 Porto, Portugal; 6 CIIMAR—Centro Interdisciplinar de Investigação Marinha e Ambiental, Universidade do Porto, 4450-208 Porto, Portugal

**Keywords:** antimicrobial peptides, piscidins, European sea bass (*Dicentrarchus labrax*), gene expression, antimicrobial activity

## Abstract

Fish rely on their innate immune responses to cope with the challenging aquatic environment, with antimicrobial peptides (AMPs) being one of the first line of defenses. Piscidins are a group of fish specific AMPs isolated in several species. However, in the European sea bass (*Dicentrarchus* *labrax*), the piscidin family remains poorly understood. We identified six different piscidins in sea bass, performed an in-depth molecular characterization and evaluated their antimicrobial activities against several bacterial and parasitic pathogens. Sea bass piscidins present variable amino acid sequences and antimicrobial activities, and can be divided in different sub groups: group 1, formed by piscidins 1 and 4; group 2, constituted by piscidins 2 and 5, and group 3, formed by piscidins 6 and 7. Additionally, we demonstrate that piscidins 1 to 5 possess a broad effect on multiple microorganisms, including mammalian parasites, while piscidins 6 and 7 have poor antibacterial and antiparasitic activities. These results raise questions on the functions of these peptides, particularly piscidins 6 and 7. Considering their limited antimicrobial activity, these piscidins might have other functional roles, but further studies are necessary to better understand what roles might those be.

## 1. Introduction

Fish are surrounded by a hostile milieu that contains a variety of microorganisms, with many of them pathogenic. Under normal conditions, fish are capable to cope with these potential invaders using a system of non-specific immune responses that confers the initial protection against pathogens [1]. Included in the repertoire of innate defenses are the antimicrobial peptides (AMPs). AMPs are found widespread throughout nature and possess strong antimicrobial and immunomodulatory activities. Due to their unique properties, AMPs have been the focus of a growing body of interest and multiple peptides are currently well characterized in several fish species [2,3,4].

Some fish species possess a particular group of amphipathic and α-helical AMPs. The first peptide belonging to this family was found in the skin secretions of the winter flounder (*Pleuronectes americanus*) and named pleurocidin [5]. Later, an amphipathic α-helical peptide was also found in the hybrid striped bass (*Morone chrysops* × *Morone saxatilis*) [6]. With the increasing interest in these fish specific AMPs, it is now known that these molecules are present in several teleost species from different families [7,8,9,10] and are generally called piscidins. Piscidins constitute a diverse group of AMPs with distinct amino acid sequences. They are produced as a prepropeptide that undergoes proteolytic cleavage to remove the signal peptide and the prodomain, resulting in a small mature peptide, usually with 18 to 26 amino acids [11,12,13,14,15,16]. However, there are reports in several species, including the white bass (*Morone chrysops*), striped bass (*Morone saxatilis*) and hybrid striped bass, of piscidins with mature peptides that range from 22 to 55 amino acids [17,18,19]. This diversity suggests that piscidins are undergoing a positive Darwinian selection and gene duplications [20,21], supporting the variety of peptides and reduced sequence identity among the piscidin family members.

The basal expression of piscidin genes is found to vary within and between fish species. These genes are constitutively expressed in tissues including the gills, skin, intestine, head kidney or spleen [8,9,16,22,23]. Several reports have shown a modulation of piscidin gene expression after infection with different pathogens [10,24,25]. Furthermore, piscidin peptides can be found in mucosal tissues at such concentrations that are lethal for pathogens [26,27]. In vitro, the capacity of these AMPs to kill different fish and mammalian pathogens has been tested, and results demonstrate that piscidins are, in fact, active against them [17,28,29,30,31,32]. Together, these evidences support the role of piscidins as peptides involved directly in the immune response against infection.

In the European sea bass (*Dicentrarchus labrax*, Moronidae), a commercially important species in aquaculture, a detailed study of the piscidin family is missing, although a 22 amino acid piscidin like peptide has been isolated and called dicentracin, and shares a high similarity with the white bass and striped bass moronecidin [33]. Furthermore, peptides identified as piscidins were detected in sea bass, using different techniques, including Western blot, ELISA, or immunohistochemistry, in tissues such as the intestine and gills [27,34]. However, while the characterization of piscidins in other fish species demonstrates the diversity and antimicrobial role of these peptides, in sea bass, the identification of the several piscidin types and a detailed study of their biological roles remains poorly explored.

In this study, we thoroughly characterize the piscidin family in sea bass, at the genomic and protein levels. The basal expression of these genes was evaluated, as well as the antimicrobial activity of piscidin mature peptides against a wide range of pathogens. Similarly to other species from the Moronidae family, sea bass piscidins are divided into different sub-groups, presenting a diverse amino acid sequence and antimicrobial activities [19]. Furthermore, sea bass piscidin mature peptides are generally larger in length (ranging from 44 to 65 amino acids), when compared to the typical piscidins (ranging from 18 to 22 amino acids), showing a degree of conservation in this family of fishes. However, further studies will be required to better understand the specific functions of these peptides, and to bring new insights about the potential of these molecules in pathogen clearance, immune defenses and possibly other functions. 

## 2. Results

### 2.1. Molecular Characterization of Sea Bass Piscidins

**Figure 1:**
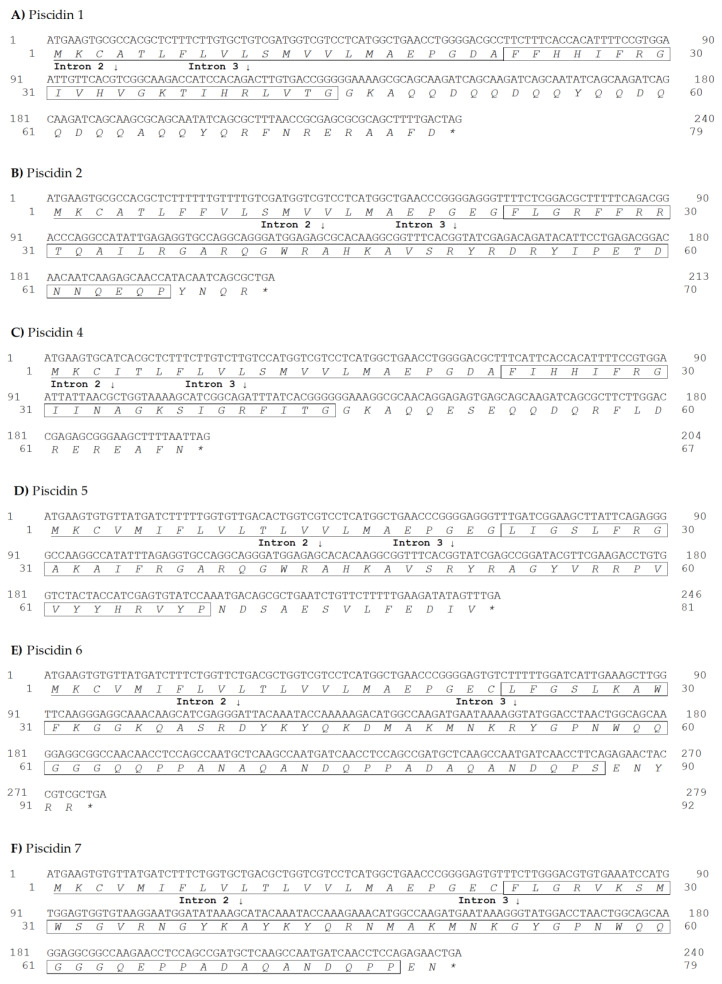
Sea bass piscidins coding DNA and amino acid sequences. Piscidin 1 (**A**), piscidin 2 (**B**), piscidin 4 (**C**), piscidin 5 (**D**), piscidin 6 (**E**) and piscidin 7 (**F**). Nucleotides are indicated in the upper row, and amino acids are indicated in italic in the lower row. Signal peptides are underlined and mature peptides are boxed. Intron positions are indicated by arrows.

Six different piscidin genes were obtained by PCR amplifications and 5’/3’ RACE using liver, intestine, gill and pyloric caeca cDNA. Piscidin genes were deposited on GenBank under accession numbers MT066191 to MT066196. Potential cleavage of sites of signal peptides and prodomains were determined using SignalP-5.0 (http://www.cbs.dtu.dk/services/SignalP/) and alignment of sea bass piscidins with peptides isolated in previous studies [17,19,33]. Molecular weights (Da) were determined, as well as the isoelectric points (pI) and net charges at pH 7. Results are shown in Table 1 and Figure 1.

**Figure 2:**
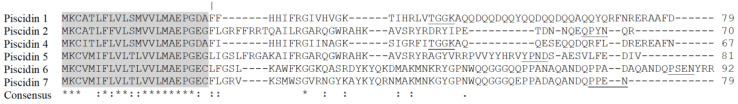
Alignment of sea bass piscidins. Signal peptides are shaded gray and predicted cleavage sites are underlined. Identical residues are denoted by (*), conserved substitutions by (:) and semi-conserved substitutions by (.).

Alignment of sea bass piscidins shows a low degree of identity of the mature peptides and prodomains, with different sizes and amino acid compositions, and a highly similar signal peptide (Figure 2). Identity scores between the different sea bass piscidins are shown in Table 2. Piscidin 1 shares a high identity with piscidin 4 (67.3%), piscidin 2 shares the highest percentage of identity with piscidin 5 (58.2%) and piscidins 6 and 7 share an identity of 64.3%.

### 2.2. Genomic Organization

**Figure 3:**
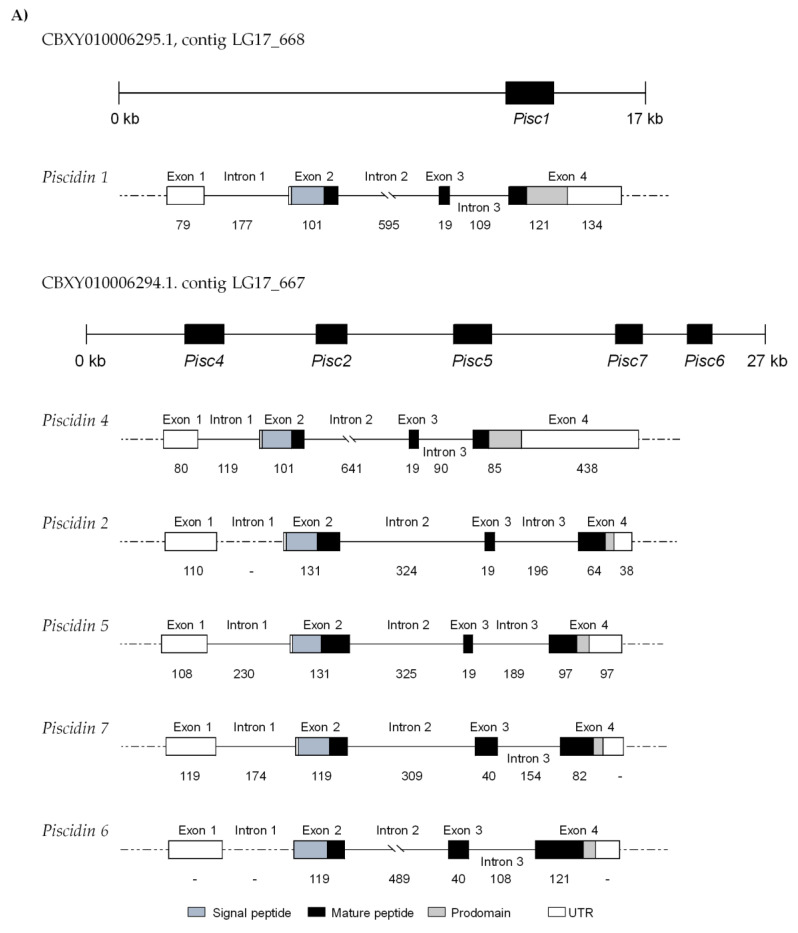

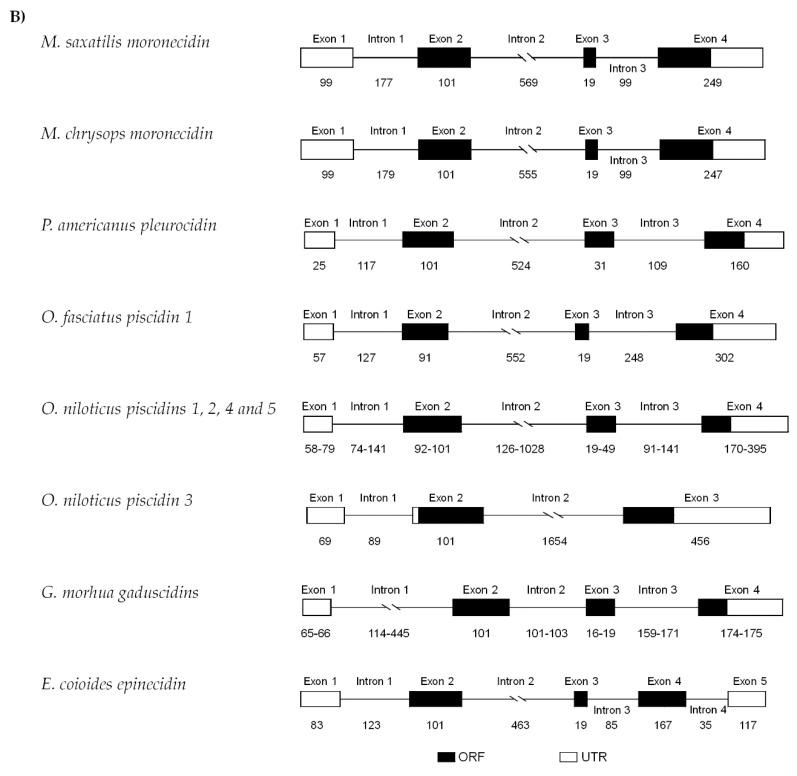
Genomic organization of sea bass piscidin genes. (**A**) Exon/intron diagram of sea bass piscidin genes and their position in the two different contigs of whole genome shotgun sequences. (**B**) Comparative view with other fish species. Exons are shown as boxes and introns as solid lines, with sizes in base pairs indicated below. Regions with unknown sizes are shown as dashed lines.

All piscidin genes were found to have a four exon/three intron structure (Figure 3A). The first exon is formed by a 5’ UTR that extends until the first nucleotide of exon 2. The signal peptides are encoded by exon 2 and the mature peptides are encoded by exons 2, 3 and 4. The prodomains are encoded by exon 4, followed by the 3’ UTR. All exon–intron boundaries follow the classical splicing motifs (GT/intron/AG). Piscidin genes show variable-sized exons, mostly the exon 4. Piscidins 1 and 4 share the same-sized exon 2 and 3 (101 and 19 bp), as well as piscidins 2 and 5 (131 and 19 bp). Piscidins 6 and 7 also show second and third exons with similar sizes (119 and 40 bp) however, the third exon is bigger when compared to the other piscidins. Comparisons with contigs available in gene databases have also shown that these genes likely share the same genetic locus, as piscidins 2, 4, 5, 6 and 7 were all found in the same contig, (CBXY010006294), with piscidin 1 in a separate contig (CBXY010006295). However, it is also likely that piscidin 1 is located upstream of piscidin 4, in total forming three clusters of 2 piscidins each, sharing higher degrees of similarity among them (Piscidin1/Piscidin4, Piscidin2/Piscidin5, and Piscidin6/Piscidin7), a hypothesis further reinforced by sequence comparisons, phylogenetic analysis and molecular modeling. 

Comparison between sea bass piscidin genes and those of other fish species shows a high similarity in terms of gene structure, with the usual organization of four exons/three introns of variable sizes (Figure 3B). However, variations from the common organization occur, particularly in Nile tilapia (*Oreochromis niloticus*) and orange spotted-grouper (*Epinephelus coioides*), that show a genomic structure comprised by three exons/two introns and five exons/four introns, respectively. Similarities in the third exon are observed between species, being the smaller exon found in piscidin genes, with sizes that range from 19 to 49 bp. The second intron is the largest among the different fish species, with variable sizes (126 to 1654 bp), with the exception of the Atlantic cod (*Gadus morhua*) gaduscidins.

### 2.3. Sequence Comparison and Phylogenetic Analysis

**Figure 4:**
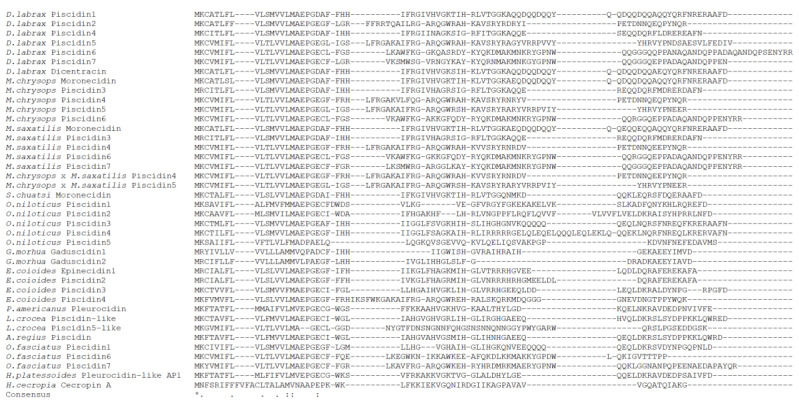
Alignment of sea bass piscidins with peptides from other fish species and with cecropin A from cecropia moth. Identical residues are denoted by (*), conserved substitutions by (:) and semi-conserved substitutions by (.).

Comparison between sea bass piscidins and peptides from other fish species shows a low degree of similarity in the mature peptides and prodomains, with the exception of piscidins that belong to other species from the Moronidae family, namely striped bass, white bass and hybrid striped bass (Figure 4). Piscidin 1 shares 97.3% of homology with dicentracin and identities between 41.6% and 97.3% with other piscidins; piscidin 2 between 41.6% and 82.3%; piscidin 4 between 45.1% and 92.9%; piscidin 5 between 36.3% and 86.7%; piscidin 6 between 23.9% and 78.8% and piscidin 7 between 37.2% and 89.4% (Table S1). 

**Figure 5:**
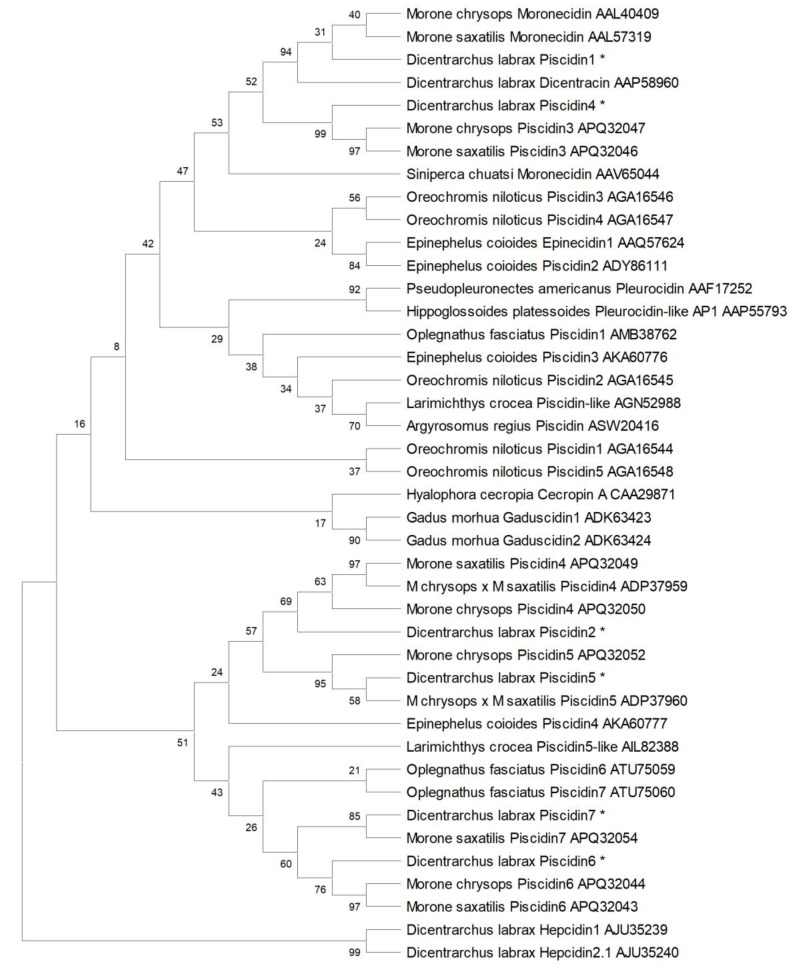
Phylogenetic analysis of piscidin peptides. The evolutionary history was inferred by using the Maximum Likelihood method and Jones–Taylor–Thornton (JTT) matrix-based model [35]. The bootstrap consensus tree inferred from 10,000 replicates is taken to represent the evolutionary history of the taxa analyzed [36]. Branches corresponding to partitions reproduced in less than 50% bootstrap replicates are collapsed. The percentage of replicate trees in which the associated taxa clustered together in the bootstrap test (10,000 replicates) are shown next to the branches [36]. Initial tree(s) for the heuristic search were obtained automatically by applying Neighbor-Join and BioNJ algorithms to a matrix of pairwise distances estimated using a JTT model. This analysis involved 42 amino acid sequences. All positions with less than 95% site coverage were eliminated. There were a total of 56 positions in the final dataset. Evolutionary analyses were conducted in MEGA X [37].

Phylogenetic analysis clusters piscidins and cecropin separated from sea bass hepcidins, another family of antimicrobial peptides (Figure 5). Among piscidins, two big clusters are evident. The first one includes a more diverse set of piscidins and piscidin-like peptides, such as moronecidin, pleurocidin, gaduscidin, epinecidin and cecropin, as well as sea bass piscidins 1 and 4. The second cluster is solely comprised of piscidins, and can be further sub-divided in two groups, separating piscidins 2 and 5 from piscidins 6 and 7. As such, we can consider that sea bass piscidins are divided into three sub-groups: piscidins 1 and 4 clusters with sea bass dicentracin, moronecidins and piscidins 3 from white (*M. saxatilis*) and striped (*M. chrysops*) basses; piscidins 2 and 5 are positioned in the same cluster with white (*M. saxatilis*), striped (*M. chrysops*), and hybrid striped (*M. saxatilis* × *M. chrysops*) basses piscidins 4 and 5; and finally, piscidins 6 and 7 are included in the same group of white (*M. saxatilis*) and striped (*M. chrysops*) basses piscidins 6 and 7. Among the two big clusters, analysis separate piscidins belonging to Moronidae species from the Atlantic cod (*G. morhua*) gaduscidins, pleurocidins from winter flounder (*P. americanus*) and American plaice (*Hippoglossoides platessoides*), cecropin A from cecropia moth (*Hyalophora cecropia*) and tilapia piscidins (*O. niloticus*).

### 2.4. Basal Expression of Sea Bass Piscidins 

**Figure 6:**
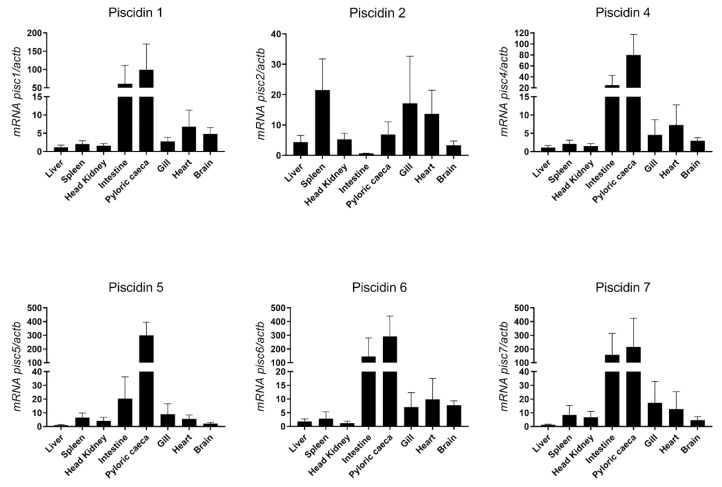
Basal expression of piscidin genes in different organs of healthy sea bass, measured by real-time PCR. Each sample was normalized to beta actin (*actb*) calculated by the comparative CT method (2^-ΔΔCT^). Values are presented as means ± standard deviation (S.D.) (*n* = 5).

Constitutive expression of piscidin genes was evaluated in different tissues of healthy sea bass, namely liver, spleen, head kidney, intestine, pyloric caeca, gill, heart and brain (Figure 6). The highest expression of most piscidin genes is observed in the intestine and pyloric caeca, with a moderate expression in the gill, heart and brain, and a low expression in the liver, spleen and head kidney. Piscidin 2, however, has a different pattern of expression, with highest expression in the spleen, gill and heart, moderate expression in the liver, head kidney, pyloric caeca and brain, and lowest expression in the intestine. 

### 2.5. Modeling of Sea Bass Piscidins

**Figure 7:**
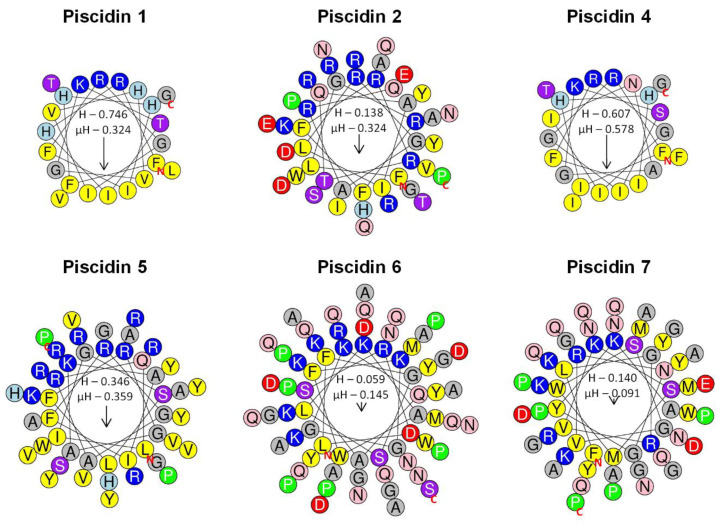
Schiffer–Edmundson helical wheel diagrams of sea bass piscidins. Piscidin 1, piscidin 2, piscidin 4, piscidin 5, piscidin 6 and piscidin 7. Positively charged residues are represented in blue circles, the negatively charged in red, the hydrophobic in yellow, the hydrophilic in purple, the amide in pink and the small residues in grey. Arrows indicate the direction of the hydrophobic moments. The red N and C represent the N-terminal and C-terminal of the peptide sequence. H represents peptide hydrophobicity, and µH represents the hydrophobic moment, which is a quantitative measure of amphipathicity.

Schiffer–Edmundson helical wheel modeling was used to predict the hydrophobic and hydrophilic regions in the secondary structure of sea bass piscidin mature peptides (Figure 7). All piscidins are predicted to form an amphipathic α-helix, with piscidin 1 and piscidin 4 showing a clear hydrophobic slant to one side, while piscidins 2, 5, 6 and 7 present most of the hydrophobic residues along one side of the helix and the hydrophilic residues along the other. Piscidin 6 and piscidin 7 likely have a more limited amphipathicity, since some hydrophilic residues might interfere with the hydrophobic side.

### 2.6. Antibacterial Activity of Sea Bass Piscidins

The antibacterial activity of piscidin mature peptides was evaluated using different Gram-negative and Gram-positive bacteria, known to cause severe diseases in aquaculture (Table 3). Piscidins 1 and 5 are highly active against almost all the bacteria tested and show the lowest MIC values that range from 2.4 ± 0.8 to 64.2 ± 2.1 µM and 1.3 ± 1.2 to 47.9 ± 6.1 µM, respectively. Piscidins 2 and 4 show a more moderate degree of activity. Piscidin 2 is capable of inhibiting the growth of many bacterial strains, but MIC values are higher than the ones observed for piscidins 1 and 5 (6.9 ± 2.8 to 146.1 ± 21.4 µM). Piscidin 4 is active only against *Photobacterium damselae* subsp. *piscicida*, *P. damselae* subsp. *damselae*, *Lactococcus garviae*, and *Streptococcus parauberis*, with values that range from 1.8 ± 1.8 to 81.3 ± 9.5 µM. On the contrary, piscidins 6 and 7 show little or no antibacterial activity against these bacteria. Nevertheless, piscidin 6 is capable of inhibiting *S. parauberis* growth (87.7 ± 123.0 µM). None of the peptides had an effect on *Aeromonas hydrophila* and *Edwardsiella tarda* growth, even at the highest concentration levels tested.

### 2.7. Antiparasitic Activity of Sea Bass Piscidins

The antiparasitic activity of sea bass piscidin mature peptides was determined using two different parasites, namely the bloodstream forms of *Trypanosoma brucei brucei* and *Leishmania infantum* promastigotes (Table 4). *T. brucei* is more susceptible to the action of the peptides, when compared to *L. infantum*. Piscidins 1, 2, 4, 5, and 7 show high activity against *T. brucei*, with low inhibitory concentration values, that range from 1.74 ± 0.13 to 7.30 ± 0.32 µM, with piscidin 2 being the most active; on the contrary, piscidin 6 is less active, presenting a IC_50_ of 56.50 ± 1.61 µM. *L. infantum* growth is also inhibited by the different piscidins, with the exception of piscidin 6. Inhibitory concentrations determined range from 5.20 ± 0.03 to 66.77 ± 0.08 µM, with piscidin 1 being the most active and piscidin 7 the less active.

## 3. Discussion

Piscidins, a fish specific antimicrobial peptide family, has been widely studied in various fish species, with proved involvement in different roles, including a direct pathogen killing action and an immunomodulatory activity. In sea bass, only a single member of this family, dicentracin, was characterized in terms of sequence and tissue expression [33]. Later, studies regarding the modulation of dicentracin expression after different stimuli became available, demonstrating that this antimicrobial peptide is triggered under harmful conditions, being an indicator of fish health [38,39]. Although previous reports already demonstrated that there are different piscidins in sea bass [27,33,34,40], the studies on the characterization and gene expression of piscidins in this fish species were focused on a single AMP, with no previous reports that describe in detail the diversity of the piscidin family in sea bass. In this work, we were able to identify six different piscidins in sea bass and expanded the piscidin coding and amino acid sequences characterized in this fish species. After comparing piscidin 1 with dicentracin, differences were observed in two amino acids of the mature peptides (Thr37/Ser37; Arg40/Lys40) and in one residue of the prodomains (Gln66/Glu66). In fact, multiple isoforms of piscidin 1 were isolated in sea bass, with differences in these particular residues (results not shown). Piscidin 1 and dicentracin share a high percentage of identity with white bass and striped bass moronecidins. Lauth et al. studied these two isoforms in the hybrid striped bass and showed the high similarities between both peptides [41]. Likewise, replacements in piscidin 1 and dicentracin mature peptides were with amino acids with similar properties, suggesting that these isoforms maintain its conformation and function [33]. All sea bass piscidins possess a similar peptide structure, formed by a signal peptide, a mature peptide and prodomain. However, while the signal peptide maintains a high degree of similarity between them, the prodomains and the active mature peptides are strikingly different. Molecular characterization, sequence alignment and phylogenetic analysis show a relationship between the genera *Dicentrarchus* and *Morone* [6,17,18,19], with respect to the number and amino acid compositions of piscine AMPs.

Piscidin family members diverge in their number and sequences. The general genomic features of these genes include a first exon constituted by a 5’ UTR, a translation initiation site positioned at exon 2 and a small sized exon 3 [9,11,14,15,42]. Previous reports showed the existence of genome clusters composed by different piscidin genes. These genes are formed by the usual four exon/three intron structure but encode different putative antimicrobial peptides, with the exception of Nile tilapia (*O. niloticus*) and grouper (*E. coioides*), that present piscidin genes with three exons/two introns and five exons/four introns, respectively [7,9,43]. During the evolutionary process, teleost fish have suffered whole genome and/or segmental duplications, leading to multiple copies of several gene families and to an enormous diversification and adaptation of species found among the Teleostean [44]. Furthermore, several genes in fish, including the ones that encodes for AMPs, are evolving rapidly and events of positive selection were already demonstrated, with a high rate of amino acid substitutions, particularly in the mature peptides [20,21,45,46]. Piscidins are no exception; in sea bass, these AMPs possess several differences, but the general structure of piscidins genes is similar among them, being found tandemly in the sea bass genome. It is possible that piscidin genes diverged from a common ancestral and, during evolution, duplications and different degrees of mutations led to the existence of multiple piscidin orthologues in sea bass.

Comparisons between sea bass and other fish species piscidins show a conserved signal peptide in terms of amino acid composition and cleavage site, with the mature peptides and prodomains sharing reduced similarities. Sea bass piscidins were also found to be formed by a high number of positive and hydrophobic residues, supporting the amphipathic nature of these AMPs. Sea bass piscidins can be divided into different sub-groups: group 1, formed by piscidins 1 and 4, both with a mature peptide of 22-aa; group 2, formed by piscidins 2 and 5, with 44- and 46-aa, respectively; and group 3, constituted by piscidins 6 and 7, with the biggest mature peptides in length, 65- and 55-aa, respectively. Salger et al. classified piscidins from white bass, striped bass and hybrid striped bass in a similar fashion: the Class I piscidins are constituted by the smaller piscidins (22-aa mature peptide), with a broad activity against different bacteria and ciliated protozoans; the Class II piscidins are formed by 44- and 46-aa mature peptides, also with varied antimicrobial activities against bacteria and parasites; and finally, the Class III piscidins are constituted by the biggest piscidins (55-aa mature peptides), showing mostly anti-protozoal activity and a reduced antibacterial activity [19]. Considering the similarities between these species, a division into different groups can also be established for sea bass peptides. The genomic structure of piscidin genes may also support the proposed division of this family, since genes from each group share the same sized exons 2 and 3. 

The overall basal expression of piscidins in sea bass resembles what was described for other teleost species. Piscidin genes and peptides are usually detected not only in sites with potential for pathogen entry, but also in immune related tissues of fish, including the intestine, spleen, head kidney, gills and skin. They can also be detected more broadly in organs such as the liver, heart or brain, depending on the piscidin and fish species [8,16,25,27,42]. Sea bass piscidin genes were detected in the organs above mentioned, being the gut the tissue with the highest basal expression levels. Assuming the antimicrobial role of piscidins, it is expected to find such peptides in mucosal tissues, such as the gastrointestinal tract and gills. Piscidin 2, homologous of white bass and striped bass piscidin 4, presents a unique pattern of expression, with the spleen and gills assuming the more abundant expression. Salger et al. also observed different levels of expression of piscidins 4 and 5 in the hybrid striped bass, being the first more abundant in the gill and the second in the intestine, showing a tissue-specific profile for these two piscidins [18,19]. Dicentracin was detected in circulating, peritoneal and head kidney leukocytes [33]. Considering the overall low constitutive expression of piscidin 1 observed in the head kidney, a hematopoietic organ in fish, it seems that this is not the main tissue presenting piscidin 1 expressing cells. Nevertheless, some kidney resident cells may express this particular piscidin, and thus it being detected by in situ hybridization techniques [33].

The amphipathic α-helical secondary structure presented by several AMPs is crucial for peptide activity. Some reports already explored piscidin conformation and activity, as well as their mode of action on pathogen membranes, using artificial membranes that mimic the natural ones and can elucidate the mechanisms underlying the effects observed on pathogenic cells [47,48,49]. In addition, Schiffer–Edmundson helical wheel diagrams are often used to predict the hydrophobic and hydrophilic regions in the secondary structure of these peptides. Using these helical wheel diagrams, other authors were able to predict the amphipathic α-helical conformation of piscidins 1 and 3 from hybrid striped bass [6,41], that was later confirmed in different studies. Piscidin 1 and 3 adopt an amphipathic α-helical structure in the presence of membrane-mimicking environments, allowing peptides to be oriented parallel to membrane surface and form pores, possibly through toroidal or in-plane diffusion mechanisms [50,51,52]. In this study, we predicted the amphipathicity of the secondary structure of sea bass piscidins. Piscidins 1 and 4, homologous of striped/white bass piscidins 1 and 3, fold into the typical structure for these peptides, constituted by an α-helix where hydrophobic residues clearly stay together in one side of the structure, in opposition to the hydrophilic ones. However, these two mature peptides share an identity of 63.6% and the most significant differences are a histidine substituted for an asparagine at position 11 and a glycine substituted for a histidine at position 17, that would probably result in different antimicrobial activities. This is in accordance to the results of Silphaduang and Noga, that evaluated protein sequences and antimicrobial capacities of piscidins 1 and 3 and observed a higher activity of piscidin 1 against a wide range of pathogens, when compared to piscidin 3 [6]. Likewise, sea bass piscidin 4 is less active against pathogens and, thus, is in accordance with previous findings. 

The structure and mode of action of white/striped bass piscidin 4 was also addressed and authors determined that this antimicrobial peptide presents an amphipathic α-helical N-terminal, while the C-terminal folds into random coils and sheets [17,18,53]. Nevertheless, this peptide may act on pathogens in a similar manner than the smaller piscidins, through binding of the N-terminal to cell membranes, then forming pores compatible to an in-plane diffusion mechanism [53]. Moreover, due to the particular structure of these two similar peptides, authors suggested that additional functions might be associated to piscidins 4 and 5 [17,19]. More specifically, piscidin 5 present a β-sheet region that is similar to known pattern recognition receptors, namely carbohydrate and lipopolysaccharide binding motifs [19]. Sea bass piscidins 6 and 7 are also predicted to be amphipathic in nature, with a reduced amphipathicity, when compared to the other piscidin family members. Salger et al. predicted a different secondary structure for these piscidins, consisting in a coil-β-sheet-coil-helix organization and showing a degree of amphipathicity [19]. However, the structure and potential roles of these atypical piscidins are not well understood. 

Although there are evident similarities between fish species that belong to the *Morone* and *Dicentrarchus* genera, specific differences between each species are also observed, with sea bass being no exception. Comparison between piscidins from *D. labrax* with *Morone* species shows variances in specific residues, that can translate into diverse antimicrobial activities. However, the main differences are observed in piscidins 5, 6 and 7. Piscidin 5 is expressed in white bass, while it was only isolated as a non-functional pseudogene in striped bass. Likewise, piscidin 7 is present in striped bass, but the authors were unable to detect it in the white bass genome [19]. In sea bass, both piscidins were isolated and detected in different tissues, with sea bass piscidin 5 presenting a prodomain constituted by 13 amino acids, contrary to what is observed for white bass. Moreover, despite similarities, sea bass piscidin 6 mature peptide shows unique features, namely a different amino acid composition between positions 14 and 17 (QARS in sea bass, KGFQ in white and striped basses), and a 10 amino acid repetition in the C-terminal of the mature peptide (ADAQANDQPS). Thus, while piscidin 6 in *Morone* species is constituted by a 55 amino acid mature peptide, in sea bass, this present an active peptide with 65 amino acids. This goes in accordance with previous findings, showing that smaller sized piscidins are usually more conserved sequence wise, whereas larger sized piscidins become much more diversified. Many duplicated genes continue under selective pressure, and while several of the duplicates usually retain their original functions, other genes are retained owing to different processes, particularly subfunctionalization, when the functions of the ancestral gene are divided among the duplicated genes, or neofunctionalization, by gaining or accumulating a novel function. Some duplicates may even become nonfunctional due to the accumulation of deleterious mutations [46]. As suggested by Salger et al., piscidins could be subjected to these evolutionary processes, resulting in a diversified family with different anti-bacterial and anti-protozoan properties [19]. 

As expected, sea bass piscidins exhibited multiple antibacterial activities, depending on the pathogen and peptide. Results of the present study show the diversity of piscidin activities, even within each sub-group. Sea bass piscidins 1 and 5, positioned into different sub groups, are the most active peptides, inhibiting the growth of almost all pathogens analyzed and presenting the lowest inhibitory concentrations. The MICs and minimal bactericidal concentrations (MBCs) of piscidins 1/moronecidins and piscidin 3 from white, striped and hybrid striped bass were previously addressed, with different outcomes. Silphaduang and Noga observed different inhibitory and bactericidal concentrations for *A. salmonicida* and *A. hydrophila*, with the first one presenting a higher resistance [6]. Furthermore, Lauth et al. observed a resistance of *A. hydrophila* when incubated with a synthetic and amidated white bass moronecidin [41]. In this study, we observed that *A. hydrophila* growth was not inhibited by any of the peptides tested, even at the highest concentration levels. Furthermore, sea bass piscidins 1, 2 and 5 presented intermediate levels of *A. salmonicida* inhibition, with no effect of piscidins 4, 6 and 7. The other bacteria tested show a diverse degree of susceptibility against synthetic piscidin peptides. *Photobacterium damselae* subsp. *piscicida* and *S. parauberis* are the most susceptible strains. On the contrary, *E. tarda* is resistant to all piscidins. A similar outcome is observed in other studies, with piscidins presenting a weak activity against *E. tarda* [25,54]. As for *P. damselae* subsp. *piscicida*, a previous study shows varied activities of hybrid striped bass piscidin 4 against different strains [17]. These differences observed between the several strains may be related with specific variations between them, translating into a diverse activity by piscidins against these bacteria [14]. The action of AMPs relies on an initial binding between them and cell membranes, through hydrophobic and electrostatic interactions. Secondary structure, charge, hydrophobicity, and amphipathic character are of most importance for peptide activity [55]. Thus, the reduced hydrophobicity and amphipathicity of piscidins 6 and 7 may also explain why these peptides present such a poor activity against the tested bacteria, when compared to the other piscidins. 

The effects of piscidins on mammalian bacteria, virus and fungi are well demonstrated [30,41,56,57]. However, the antimicrobial activity against mammalian parasites is not well explored. Thus, the anti-parasitic activity of sea bass piscidins was evaluated against *T. brucei brucei* and *L. infantum*, parasites known to infect mammalian species, including humans, mainly in developing tropical countries [58]. To our knowledge, this is the first report that explores the action of fish antimicrobial molecules against these particular parasites. We demonstrate that piscidins 1 to 5 are highly effective in inhibiting the growth of both parasites in vitro, with piscidin 6 and 7 presenting the lowest anti-parasitic capacity. According to the findings of Salger et al., Class III piscidins present a reduced action against bacteria, but a strong anti-protozoal activity [19]. In this study, this was not observed, with the smaller peptides being more active against *T. brucei* and *L. infantum* when compared to sea bass piscidins 6 and 7. Still, the action of these synthetic piscidins was not tested on fish parasites, and their antiparasitical activity may be more effective on these microorganisms, while the other smaller peptides have a broader effect on different pathogens.

## 4. Materials and Methods 

### 4.1. Animals

European sea bass (*Dicentrarchus labrax*), with an average weight of 30 g, were provided by a commercial fish farm (Sonríonansa S.L., Pesués, Cantabria, Spain). Fish were kept at the fish holding facilities of the Instituto de Ciências Biomédicas Abel Salazar (ICBAS), Porto, Portugal, in 110-L recirculating sea water (28‰ salinity) tanks at 23 °C, with a 13 h/11 h light-dark cycle, and fed daily to satiation with commercial fish feed. Before each treatment, fish were anesthetized with ethylene glycol monophenyl ether (2-phenoxyethanol, 0.3 mL/L; Merck, Algés, Portugal). All animal experiments were carried out in strict compliance with national and international animal use ethics guidelines, approved by the animal welfare and ethic committees of ICBAS (P293/2019/ORBEA, 05/04/2019), and conducted by experienced and trained Federation of European Laboratory Animal Science Associations Category C investigators.

### 4.2. Isolation of Sea Bass Piscidins 

Pairs of oligonucleotide PCR primers were designed according to conserved regions of dicentracin mRNA sequence from sea bass and piscidins from other fish species, sea bass expressed sequence tags (ESTs) and whole-genome shotgun sequences (WGSS) available in the National Center for Biotechnology Information nucleotide database (http://www.ncbi.nlm.nih.gov). cDNA preparations from whole intestine, liver, gill and pyloric caeca were used in PCR amplifications. PCR products were run on 1.2% agarose gels, and relevant fragments purified with the NZYGelpure kit (NZYtech, Lisbon, Portugal), cloned into pCR™2.1-TOPO^®^ vectors, propagated in One Shot^®^ Mach1™-T1R competent cells (Invitrogen, Life Technologies, Carlsbad, CA, USA), and sent for sequencing (GATC, A Eurofins Genomics Company, Ebersberg, Germany). Both strands were sequenced, and chromatograms were analyzed in FinchTV (Geospiza, Seattle, WA, USA) and assembled using Multalin (http://multalin.toulouse.inra.fr/multalin/). The Schiffer–Edmundson helical wheel diagrams, hydrophobicity and hydrophobic moment were determined using HeliQuest (http://heliquest.ipmc.cnrs.fr/) [59].

### 4.3. Genomic Organization 

Genomic DNA was isolated from sea bass red blood cells, using the NZY Blood gDNA Isolation kit (NZYtech, Lisbon, Portugal). Quantification was performed using a NanoDrop 1000 spectrophotometer (Thermo Fisher Scientific, Waltham, MA, USA) and quality was checked by agarose gel electrophoresis. Two micrograms of genomic DNA were amplified by PCR with the primers based on the previously obtained cDNA sequences, with the following cycling profile: 94 °C for 5 min, 30 cycles of 94 °C for 60 s, 59 °C for 60 s, 72 °C for 60 s and a final step of 72 °C for 5 min. Several PCR products were purified, cloned, and sent for sequencing. Comparisons were made between cDNA and genomic DNA to assess the similarity of the coding regions and to identify intron/exon boundaries. A comparison between the genomic sequences of sea bass piscidins with those of other species was also made, using the sequences identified with the following GenBank accession numbers and previous studies: *M. chrysops* moronecidin (AF394243), *M. saxatilis* moronecidin (AF394244); *Pseudopleuronectes americanus* pleurocidin (AF210241); *Oplegnathus fasciatus* piscidin-1 (KT354978); *Oreochromis niloticus* piscidin1 to 5 [9]; *Gadus morhua* gaduscidins/piscidins [42]; *Epinephelus coioides* epinecidin-1 [7].

### 4.4. Amplification of 5’ and 3’ Flanking Regions

The 5’ and 3’ RACE were carried out using the 5’/3’ RACE Kit, 2nd Generation (Roche Applied Science, Amadora, Portugal) according to the manufacturer’s instructions. Conditions for PCR were as follows: 94 °C for 2 min, 94 °C for 15 s, 59 °C for 30 s, 72 °C for 40 s, for 10 cycles; 94 °C for 15 s, 59 °C for 30 s, 72 °C for 40 s (plus 20 s/cycle), for 25 cycles, with a final elongation at 72 °C for 7 min. When necessary, a second PCR amplification was performed using the same conditions for an additional 30 cycles. To increase sequence coverage and obtain possible promoter regions, amplifications of genomic DNA were performed using the Universal Genome Walker Kit (Clontech, MountainView, CA, USA), according to the manufacturer’s instructions. Amplification products were run on agarose gels, relevant fragments purified, cloned, and sequenced as previously described.

### 4.5. Alignment and Phylogenetic Analysis

Alignments of the amino acid sequences of the piscidin predicted proteins were performed using MUSCLE from MEGA X [37]. A phylogenetic tree was constructed using the Maximum Likelihood method, with the Jones–Taylor–Thornton (JTT) model [35], Nearest-Neighbor-Interchange heuristic model, partial deletion of gaps, and 10,000 bootstrap replications. Sequences used for comparisons and phylogenetic trees and their accession numbers were as follows: *D. labrax* WGSS (CBXY010006294 and CBXY010006295); *D. labrax* dicentracin (AAP58960); *Siniperca chuatsi* moronecidin (AAV65044); *Oreochromis niloticus* piscidins 1 to 5 (AGA16544, AGA16545, AGA16546, AGA16547 and AGA16548); *Gadus morhua* gaduscidin 1 and 2 (ADK63423 and ADK63424); *Epinephelus coioides* epinecidin 1 (AAQ57624); *E. coioides* piscidins 2, 3 and 4 (ADY86111, AKA60776 and AKA60777); *Pseudopleuronectes americanus* pleurocidin (AAF17252); *M. chrysops* moronecidin (AAL40409); *M. chrysops* piscidins 3 to 6 (APQ32047, APQ32050, APQ32052 and APQ32044); *M. saxatilis* moronecidin (AAL57319); *M. saxatilis* piscidins 3, 4, 6 and 7 (APQ32046, APQ32049, APQ32043 and APQ32054); *M. chrysops* × *M. saxatilis* piscidins 4 and 5 (ADP37959 and ADP37960); *Larimichthys crocea* piscidin-like (AGN52988); *L. crocea* piscidin5-like (AIL82388); *Argyrosomus regius* piscidin (ASW20416); *Oplegnathus fasciatus* piscidins 1, 6 and 7 (AMB38762, ATU75059 and ATU75060); *Hippoglossoides platessoides* pleurocidin-like AP1 (AAP55793); *Hyalophora cecropia* cecropin A (CAA29871); *D. labrax* hepcidin 1 (AJU35239); *D. labrax* hepcidin 2 variant 1 (AJU35240). Identity scores between the different piscidin peptides were determined using Sequence Identity And Similarity (SIAS) software (http://imed.med.ucm.es/Tools/sias.html). 

### 4.6. RNA Isolation and cDNA Synthesis

Total RNA was isolated from tissues with the NZY Total RNA Isolation Kit (NZYTech, Lisboa, Portugal), according to the manufacturer’s instructions. Total RNA quantification was performed using a NanoDrop 1000 spectrophotometer (Thermo Scientific), and quality was assessed by running the samples in an Experion Automated Electrophoresis Station (Bio-Rad, Hercules, CA, USA). For all samples, 1.25 µg of each were converted to cDNA using the NZY First-Strand cDNA Synthesis Kit (NZYTech, Lisboa, Portugal), according to the manufacturer’s protocol.

### 4.7. Basal Expression of Sea Bass Piscidins 

Several tissues from five healthy sea bass were collected for RNA isolation and cDNA synthesis, as previously described. Relative levels of piscidin mRNAs were quantified by real-time PCR analysis using an CFX96™ Real-Time PCR Detection System (Bio-Rad). One microliter of each cDNA sample was added to a reaction mix containing 10 µL iTaq™ Universal SYBR^®^ Green Supermix (Bio-Rad), 7 µL of _dd_H20, and 250 nM of each primer (Table S2), making a total volume of 20 µL per reaction. The cycling profile was as follows: 95 °C for 3.5 min, 40 cycles of 95 °C for 20 s, and 59 °C for 20 s. Samples were prepared in duplicates, a melting curve was generated for every PCR product to confirm the specificity of the assays, and a dilution series was prepared to check the efficiency of the reactions. Beta actin (*actb*) was used as the housekeeping gene. The comparative CT method (2^-ΔΔCT^ method) based on cycle threshold values was used to analyze gene expression levels.

### 4.8. Antibacterial Activity of Sea Bass Piscidin Peptides

The biological activity of sea bass piscidin mature peptides was studied by determining their antimicrobial properties. Synthetic piscidin peptides were based on the predicted coding sequences and were commercially produced (NZYtech, Lisbon, Portugal) with an additional C-terminal amidation (Table S3). Peptides were incubated in serial dilutions with ten bacterial strains, known to cause severe diseases in aquaculture: *Photobacterium damselae* subsp. *piscicida* (DSM 22834), *P. damselae* subsp. *damselae* (DSM 7482), *Vibro anguillarum* (DSM 21597), *V. alginolyticus* (DSM 2171), *Aeromonas salmonicida* subsp. *salmonicida* (DSM 19634), *A. hydrophila* subsp. *hydrophila* (DSM 30187), *Yersinia ruckeri* (DSM 18506), *Edwardsiella tarda* (DSM 30052), *Lactococcus garviae* (DSM 20684) and *Streptococcus parauberis* (DSM 6631). In short, 1 × 10^8^ bacteria per milliliter were incubated in optimal growth conditions with the peptide in flat-bottom 96-well plates, in a final volume of 100 µL, and OD was read at 600 nm in a plate reader after 24 h of incubation. Wells with no added peptide were used as controls, and wells without bacteria were used as blanks. The minimal inhibitory concentration (MIC) was determined as the lowest concentration of piscidin that reduced 50% of bacterial growth when compared to the controls. The MICs reported correspond to representative results from two independent experiments.

### 4.9. Antiparasitic Activity of Sea Bass Piscidin Peptides

The anti-protozoal activity of sea bass piscidins was evaluated using two parasites: *Trypanosoma brucei brucei* bloodstream forms (strain Lister 427) and *Leishmania infantum* promastigotes (strain MHOM/MA/67/ITMAP-263). Parasites were incubated in optimal growth conditions with serial dilutions of the different peptides in flat-bottom 96-well plates, in a final volume of 200 µL, during 72 h. Cell metabolic activity was measured by resazurin reduction. Briefly, 20 µL of resazurin (50 µM) were added and incubated for 4 h. The fluorescence of resorufin, resulting from resazurin reduction by metabolically active cells, was measured at λ_ex_ = 544 nm and λ_em_ = 590 nm in Synergy 2 (BioTek, Winooski, Vermont, USA). Cultures with no added peptide were used as negative controls and cultures with the reference drugs, namely pentamidine (10 nM) and miltefosine (40 µM) were used as positive controls for *T. brucei* and *L. infantum* inhibition assays, respectively. Wells with culture medium were used as blanks. Anti-parasitic effect was evaluated by the determination of IC_50_ value (concentration required to inhibit growth in 50%) and calculated by non-linear regression analysis using GraphPad Prism version 8.1.1 for Windows (GraphPad Software, San Diego, CA, USA). The IC_50_ reported correspond to representative results from two independent experiments.

## 5. Conclusions

We characterized in sea bass a diverse group of piscidins, closely related with species from the genus *Morone*. This may suggest that piscidins belonging to the Moronidae family have evolved to a specialized family of antimicrobial molecules, perhaps with distinct functions, asides the wide range of activity against several pathogens. Thus, further studies are required to understand the roles of these molecules, particularly piscidins 6 and 7. Although described as piscidins, these two peptides present a limited antimicrobial activity, which opens the possibility of them being involved in other immune or non-immune mechanisms, which would not be uncommon, as there are other antimicrobial peptides that present several functions.

## Figures and Tables

**Table 1:** Characterization of sea bass piscidin genes and peptides.

	Piscidin1	Piscidin2	Piscidin4	Piscidin5	Piscidin6	Piscidin7
Accession number	MT066191	MT066192	MT066193	MT066194	MT066195	MT066196
ORF (bp) ^1^	240	213	204	246	279	240
5’ UTR (bp) ^2^	80	111	81	109	-	-
3’ UTR (bp) ^2^	134	38	438	97	-	120
Prepropeptide (aa) ^3^	79	70	67	81	92	79
Signal peptide (aa)	22	22	22	22	22	22
Mature peptide (aa)	22	44	22	46	65	55
Prodomain (aa)	35	4	23	13	5	2
Molecular weight (Da)	2571	5362	2454	5406	7182	6174
Isoelectric point (pI)	14.00	11.82	14.00	12.03	10.34	10.52
Net charge (at pH 7)	4.4	7.1	4.2	12.2	5.0	6.0

^1^ Open reading frame; ^2^ 5’ and 3’ untranslated regions; ^3^ Piscidin full length amino acid (aa) sequence.

**Table 2:** Identity scores of full length sea bass piscidin amino acid sequences.

	Piscidin1	Piscidin2	Piscidin4	Piscidin5	Piscidin6	Piscidin7
Piscidin1		37.8%	67.3%	30.6%	25.5%	33.7%
Piscidin2	37.8%		48.0%	58.2%	28.6%	43.9%
Piscidin4	67.3%	48.0%		36.7%	27.6%	34.7%
Piscidin5	30.6%	58.2%	36.7%		38.8%	48.0%
Piscidin6	25.5%	28.6%	27.6%	38.8%		64.3%
Piscidin7	33.7%	43.9%	34.7%	48.0%	64.3%	

Identity scores were determined using the SIAS software, with BLOSUM62 scoring matrix and considering the length of multiple sequence alignment.

**Table 3:** Antibacterial activity of sea bass synthetic piscidins.

Bacteria	MIC (µM)					
Gram-Negative	Piscidin1	Piscidin2	Piscidin4	Piscidin5	Piscidin6	Piscidin7
*P. damselae* subsp. *piscicida*	7.4 ± 1.6	9.1 ± 1.7	29.1 ± 12.0	3.3 ± 0.1	N.A.	N.A.
*P. damselae* subsp. *damselae*	15.0 ± 0.2	105.8 ± 22.1	81.3 ± 9.5	14.8 ± 2.0	N.A.	N.A.
*V. anguillarum*	16.3 ± 0.6	146.1 ± 21.4	N.A.	10.1 ± 2.7	N.A.	N.A.
*V. alginolyticus*	34.8 ± 0.3	66.2 ± 2.0	N.A.	36.9 ± 2.3	N.A.	N.A.
*A. salmonicida* subsp. *salmonicida*	64.2 ± 2.1	67.7 ± 0.2	N.A.	47.9 ± 6.1	N.A.	N.A.
*A. hydrophila* subsp. *hydrophila*	N.A.	N.A.	N.A.	N.A.	N.A.	N.A.
*Y. ruckeri*	48.4 ± 3.8	137.6 ± 3.1	N.A.	25.4 ± 1.8	N.A.	N.A.
*E. tarda*	N.A.	N.A.	N.A.	N.A.	N.A.	N.A.
**Gram-positive**						
*L. garviae*	33.9 ± 3.8	N.A.	48.8 ± 40.1	42.3 ± 16.4	N.A.	N.A.
*S. parauberis*	2.4 ± 0.8	6.9 ± 2.8	1.8 ± 1.8	1.3 ± 1.2	87.7 ± 123.0	N.A.

Growth inhibition of *P. damselae* spp. *piscicida*, *P. damselae* spp. *damselae*, *V. anguillarum*, *V. alginolyticus*, *A. salmonicida*, *A. hydrophila*, *Y. ruckeri*, *E. tarda*, *L. garviae* and *S. parauberis* incubated for 24 h with serial dilutions of the synthetic piscidin putative mature peptides (up to 200 µM). The MIC was determined as the lowest concentration of piscidin that reduced 50% of bacterial growth when compared to the controls. Results are representative of two separate experiments and shown as means ± SD. N.A. indicates that piscidin was not active against the bacteria tested at the highest concentration tested.

**Table 4:** Antiparasitic activity of sea bass synthetic piscidins.

IC_50_ (µM)						
	Piscidin1	Piscidin2	Piscidin4	Piscidin5	Piscidin6	Piscidin7
*T. brucei brucei*	3.88 ± 0.11	1.74 ± 0.13	3.24 ± 0.16	4.42 ± 0.12	56.50 ± 1.61	7.30 ± 0.32
*L. infantum*	5.20 ± 0.03	5.52 ± 0.26	8.34 ± 0.81	7.24 ± 0.61	N.A.	66.77 ± 0.08

*T. brucei brucei* and *L. infantum* were incubated for 72 h with serial dilutions of the synthetic piscidin putative mature peptides (up to 100 µM). The IC_50_ was determined as the lowest concentration of piscidin that inhibited 50% of parasite growth when compared to the controls. Results are representative of two separate experiments and shown as means ± SD. N.A. indicates that piscidin was not active against the parasites tested at the highest concentration tested.

## Supplementary Materials

Supplementary materials can be found at https://www.mdpi.com/1422-0067/21/13/4613/s1.
