# Supplementary material for: The Diverse Piscidin Repertoire of the European Sea Bass (Dicentrarchus labrax): Molecular Characterization and Antimicrobial Activities
Source: Int J Mol Sci. 2020 Jun 29;21(13):4613. doi: 10.3390/ijms21134613 (PMC7369796; doi:10.3390/ijms21134613)
Supplement: Supplementary file 1 [file ijms-21-04613-s001.zip › Supplementary files/Table S1.docx]

**Table S1.** Identity scores of full length sea bass piscidin amino acid sequences with peptides from other fish species.

| **Family** | **Species** | **Piscidin** | **Sea bass piscidins** | | | | | |
| --- | --- | --- | --- | --- | --- | --- | --- | --- |
|  |  |  | **Piscidin 1** | **Piscidin 2** | **Piscidin 4** | **Piscidin 5** | **Piscidin 6** | **Piscidin 7** |
| Moronidae  Moronidae  Moronidae  Moronidae  Sinipercidae Cichlidae  Gadidae  Serranidae  Pleuronectidae  Sciaenidae  Sciaenidae [Oplegnathidae](https://en.wikipedia.org/wiki/Oplegnathidae)  Pleuronectidae | *D. labrax*  *M. chrysops*  *M. chrysops*  *M. chrysops*  *M. chrysops*  *M. chrysops*  *M. saxatilis*  *M. saxatilis*  *M. saxatilis*  *M. saxatilis*  *M. saxatilis*  *M. chrysops_x_M_saxatilis*  *M. chrysops_x_M_saxatilis*  *S. chuatsi*  *O. niloticus*  *O. niloticus*  *O. niloticus*  *O. niloticus*  *O. niloticus*  *G. morhua*  *G. morhua*  *E. coioides*  *E. coioides*  *E. coioides*  *E. coioides*  *P. americanus*  *L. crocea*  *L. crocea*  *A. regius*  *O. fasciatus*  *O. fasciatus*  *O. fasciatus*  *H. platessoides* | Dicentracin  Moronecidin  Piscidin 3  Piscidin 4  Piscidin 5  Piscidin 6  Moronecidin  Piscidin 3  Piscidin 4  Piscidin 6  Piscidin 7  Piscidin 4  Piscidin 5  Moronecidin  Piscidin 1  Piscidin 2  Piscidin 3  Piscidin 4  Piscidin 5  Gaduscidin 1  Gaduscidin 2  Epinecidin 1  Piscidin 2  Piscidin 3  Piscidin 4  Pleurocidin  Piscidin-like  Piscidin5-like  Piscidin  Piscidin 1  Piscidin 6  Piscidin 7  Pleurocidin-like AP1 | 97.3%  97.3%  69.9%  46.9%  45.1%  44.2%  97.3%  69.9%  47.8%  44.2%  44.2%  47.8%  45.1%  69.0%  41.6%  50.4%  69.0%  55.8%  41.6%  42.5%  41.6%  57.5%  56.6%  57.5%  45.1%  45.1%  56.6%  43.4%  54.9%  58.4%  44.2%  49.6%  48.7% | 50.4%  50.4%  50.4%  82.3%  70.8%  46.9%  50.4%  50.4%  78.8%  46.0%  51.3%  78.8%  70.8%  49.6%  50.4%  43.4%  47.8%  41.6%  45.1%  43.4%  46.0%  53.1%  54.9%  52.2%  64.6%  43.4%  45.1%  43.4%  46.0%  53.1%  49.6%  51.3%  41.6% | 69.9%  67.3%  92.9%  47.8%  51.3%  45.1%  69.0%  92.9%  52.2%  45.1%  45.1%  52.2%  51.3%  75.2%  50.4%  50.4%  64.6%  49.6%  49.6%  48.7%  51.3%  69.0%  61.9%  57.5%  48.7%  53.1%  60.2%  50.4%  60.2%  62.8%  48.7%  46.9%  55.8% | 45.1%  44.2%  45.1%  69.0%  86.7%  53.1%  44.2%  45.1%  69.0%  52.2%  53.1%  69.0%  86.7%  46.0%  43.4%  39.8%  45.1%  36.3%  42.5%  36.3%  37.2%  49.6%  50.4%  46.9%  58.4%  45.1%  46.0%  46.0%  46.9%  46.9%  45.1%  54.0%  44.2% | 35.4%  34.5%  34.5%  45.1%  44.2%  77.9%  34.5%  34.5%  45.1%  78.8%  73.5%  45.1%  44.2%  38.9%  30.1%  28.3%  34.5%  23.9%  32.7%  23.9%  26.5%  34.5%  34.5%  35.4%  39.8%  33.6%  38.1%  38.9%  36.3%  37.2%  53.1%  57.5%  33.6% | 46.9%  46.0%  46.9%  55.8%  51.3%  81.4%  46.0%  46.9%  54.9%  81.4%  89.4%  54.9%  51.3%  49.6%  44.2%  38.9%  48.7%  38.9%  45.1%  37.2%  39.8%  46.9%  46.0%  46.9%  50.4%  44.2%  51.3%  51.3%  49.6%  50.4%  61.1%  67.3%  43.4% |

Identity scores were determined using the SIAS software, with BLOSUM62 scoring matrix and considering the length of multiple sequence alignment.
