# Supplementary material for: The Diverse Piscidin Repertoire of the European Sea Bass (Dicentrarchus labrax): Molecular Characterization and Antimicrobial Activities
Source: Int J Mol Sci. 2020 Jun 29;21(13):4613. doi: 10.3390/ijms21134613 (PMC7369796; doi:10.3390/ijms21134613)
Supplement: Supplementary file 1 [file ijms-21-04613-s001.zip › Supplementary files/Table S2.docx]

**Table S2.** Primers used for gene expression analysis.

|  | FOR (5’→3’) | REV (5’→3’) |
| --- | --- | --- |
| *actb* | CAGAAGGACAGCTACGT | GTCATCTTCTCCCTGTTGGC |
| *pisc1* | TCGTCCTCATGGCTGAACC | GCGGTTAAAGCGCTGATATTG |
| *pisc2* | TCGTCCTCATGGCTGAACC | GGTTGCTCTTGATTGTTGTCCG |
| *pisc4* | TCGTCCTCATGGCTGAACC | GGAGCTGTGTTGAAGTTGAGT |
| *pisc5* | TCGTCCTCATGGCTGAACC | CACAGGTCTTCGAACGTATCC |
| *pisc6* | TCGTCCTCATGGCTGAACC | TCAGCGACGGTAGTTCTCTG |
| *pisc7* | TGGGACGTGTGAAATCCATGT | TCAGTTCTCTGGAGGTTGATC |
