# Supplementary material for: The Diverse Piscidin Repertoire of the European Sea Bass (Dicentrarchus labrax): Molecular Characterization and Antimicrobial Activities
Source: Int J Mol Sci. 2020 Jun 29;21(13):4613. doi: 10.3390/ijms21134613 (PMC7369796; doi:10.3390/ijms21134613)
Supplement: Supplementary file 1 [file ijms-21-04613-s001.zip › Supplementary files/Table S3.docx]

**Table S3.** Synthetic piscidin mature peptides.

| Piscidin1 | FFHHIFRGIVHVGKTIHRLVTG-NH_2_ |
| --- | --- |
| Piscidin2 | FLGRFFRRTQAILRGARQGWRAHKAVSRYRDRYIPETDNNQEQP-NH_2_ |
| Piscidin4 | FIHHIFRGIINAGKSIGRFITG-NH_2_ |
| Piscidin5 | LIGSLFRGAKAIFRGARQGWRAHKAVSRYRAGYVRRPVVYYHRVYP-NH_2_ |
| Piscidin6 | LFGSLKAWFKGGKQASRDYKYQKDMAKMNKRYGPNWQQGGGQQPPANAQANDQPPADAQANDQPS-NH_2_ |
| Piscidin7 | FLGRVKSMWSGVRNGYKAYKYQRNMAKMNKGYGPNWQQGGGQEPPADAQANDQPP-NH_2_ |

Piscidins were commercially synthetized with an additional C-terminal amidation.
